# Survey and Characterization of Jingmen Tick Virus Variants

**DOI:** 10.3390/v11111071

**Published:** 2019-11-17

**Authors:** Ender Dinçer, Sabri Hacıoğlu, Sırrı Kar, Nergis Emanet, Annika Brinkmann, Andreas Nitsche, Aykut Özkul, Yvonne-Marie Linton, Koray Ergünay

**Affiliations:** 1 Research and Application Center, Advanced Technology Education, Mersin University, Mersin 33110, Turkey; enderdin@gmail.com; 2 Department of Virology, Faculty of Veterinary Medicine, Ankara University, Ankara 06110, Turkey; saabrii@hotmail.com (S.H.);; 3 Department of Biology, Namık Kemal University, Tekirdağ 33110, Turkey; sirrikar@yahoo.com; 4 Department of Microbiology and Immunology and Galveston National Laboratory, University of Texas Medical Branch, Galveston, GX 77555, USA; 5 Virology Unit, Department of Medical Microbiology, Faculty of Medicine, Hacettepe University, Ankara 06100, Turkey; nergisemanet@gmail.com; 6 Center for Biological Threats and Special Pathogens 1 (ZBS-1), Robert Koch Institute, 13352 Berlin, Germany; BrinkmannA@rki.de (A.B.); NitscheA@rki.de (A.N.); 7 Department of Entomology, Smithsonian Institution-National Museum of Natural History, Washington, DC 20560, USA; 8 Walter Reed Biosystematics Unit, Smithsonian Institution Museum Support Center, Suitland, MD 20746, USA

**Keywords:** jingmen, tick, flavivirus, Anatolia, turkey

## Abstract

We obtained a Jingmen tick virus (JMTV) isolate, following inoculation of a tick pool with detectable Crimean-Congo hemorrhagic fever virus (CCHFV) RNA. We subsequently screened 7223 ticks, representing 15 species in five genera, collected from various regions in Anatolia and eastern Thrace, Turkey. Moreover, we tested specimens from various patient cohorts (*n* = 103), and canine (*n* = 60), bovine (*n* = 20) and avian specimens (*n* = 65). JMTV nucleic acids were detected in 3.9% of the tick pools, including those from several tick species from the genera *Rhipicephalus* and *Haemaphysalis*, and *Hyalomma marginatum*, the main vector of CCHFV in Turkey. Phylogenetic analysis supported two separate clades, independent of host or location, suggesting ubiquitous distribution in ticks. JMTV was not recovered from any human, animal or bird specimens tested. Near-complete viral genomes were sequenced from the prototype isolate and from three infected tick pools. Genome topology and functional organization were identical to the members of Jingmen group viruses. Phylogenetic reconstruction of individual viral genome segments and functional elements further supported the close relationship of the strains from Kosovo. We further identified probable recombination events in the JMTV genome, involving closely-related strains from Anatolia or China.

## 1. Introductıon

Ticks (class *Arachnida*, subclass *Acari*) are hematophagous ectoparasites of vertebrates, acting as biological vectors responsible for the transmission of several viruses to humans and animals [1]. Many tick-borne viruses, such as Severe Fever with Thrombocytopenia virus (SFTSV) and Crimean-Congo Hemorrhagic Fever virus (CCHFV), are emerging or re-emerging pathogens, posing global public and animal health threats. Viruses vectored by ticks are diverse and classified in a number of viral families [2]. However, the full range of tick-borne viral agents is unknown and deep sequencing techniques have unearthed a vast diversity of viruses in ticks, including several novel strains and new viral species [3]. Despite several isolates appearing as commensal, the pathogenic potential and outcomes in vertebrate infections have not been explored in many viruses [3,4].

Jingmen tick virus (JMTV), initially detected from ticks in the Jingmen region of Hubei province in China [5], is among the recently described tick-associated viruses. Subsequent reports have described closely-related virus genomes in ticks and cattle from Brazil (Mogiana tick virus, MGTV), and in ticks from China [6,7,8,9,10]. The JMTV comprises a multicomponent genome composed of four segments [5]. Genome segments 1 and 3 encode the non-structural proteins, which are genetically and functionally related to the flavivirus NS3 and NS5 proteins [5,11]. The remaining segments encoding the structural proteins are unique and likely to have originated from uncharacterized ancestral viruses. Reports have further documented genetically-similar viruses in mosquitoes and insects from America, Africa and Asia [11,12,13]. Together, these viruses are currently considered as a separate and diverse group, tentatively called the Jingmen virus group in the family *Flaviviridae* [11].

The detection of a JMTV variant in a red colobus monkey in Uganda demonstrated primate infections in nature and highlighted the potential relevance of these viruses to animal and human health [11]. The discovery of individuals co-infected with CCHFV and JMTV in Kosovo further supported the possibility of human exposure through shared tick vectors with implications on CCHFV clinical progression [14]. Finally, a direct association of JMTV with human disease was documented in China, where the virus was detected in skin biopsies and blood as well as detached ticks from individuals with local and systemic symptoms, which could be severe in particular cases [15]. Moreover, another JMTV-like virus, named Alongshan virus (ALSV), was isolated in patients with febrile disease, in northeastern China [16]. ALSV was also detected in ticks from southeastern Finland, without documented human cases [17]. It appears that JTMV and related viruses are ubiquous, globally distributed tick-associated viruses capable of infecting a wide range of animal hosts and causing symptomatic infections in humans. Therefore, it is imperative to investigate these viruses and their potential impact in infections with unknown etiology in regions endemic for tick-borne infections.

Turkey is located in the Asia Minor and Eastern Thrace region of the Balkan Peninsula and maintains a natural transmission zone for vector-borne infections between Asia, Africa and Europe. Diverse ecological and climatic conditions observed in Anatolia and Thrace provides suitable habitats for several tick species [18]. Many tick-borne infections have been documented in Turkey, causing significant economic burden and public health problems [19]. The most prominent tick-borne virus is CCHFV, which emerged in 2002 and spread through Anatolia, with 8742 cases and mortality of 0.2%–0.88% reported during 2008–2017 (https://hsgm.saglik.gov.tr/tr/zoonotikvektorel-kkka/zoonotikvektorel-kkka-istatistik). Furthermore, we have detected Tamdy orthonairovirus, chuviruses, flavi-like viruses, rhabdoviruses and novel phleboviruses in ticks from various regions of Turkey, mostly with unexplored human or animal health impact [4,20,21]. This study was carried out to investigate JMTV in ticks, their common vertebrate hosts, and in human infections with CCHFV infections or unidentified etiology.

## 2. Materıals and Methods

### 2.1. Ethical Statement

Animal sera were obtained at local veterinary clinics and animal shelters or during tick removal, with full compliance with the national regulations on the operation and procedure of animal experiments ethics committees (Regulation No. 26220, date: July 09, 2006) and approved study protocols by Ankara University local ethics committee (AULEC/201-96-346). The tick specimens included in the study comprised field-collected host-seeking or ambusher ticks and those removed from domesticated animals, performed with informed consent and cooperation of the owners or caretakers, for which local or regional ethics committee approval was not required. Stored human sera and cerebrospinal fluid specimens were included for testing with previous approval from local or institutional ethics committees (Hacettepe University non-interventional clinical research ethics board, 16.03.2012/FON.12/05-5; Ankara training and research hospital ethics board, 13.07.2011/0426). Collection and testing of avian specimens were undertaken with approval from the Republic of Turkey ministry of forestry and water affairs, general directorate of nature conservation and national parks (20.04.2016/86035) and Ankara University animal experiments local ethics committee (10.02.2016/2016-3-22).

### 2.2. Tick Collection and Processing

**Figure 1:**
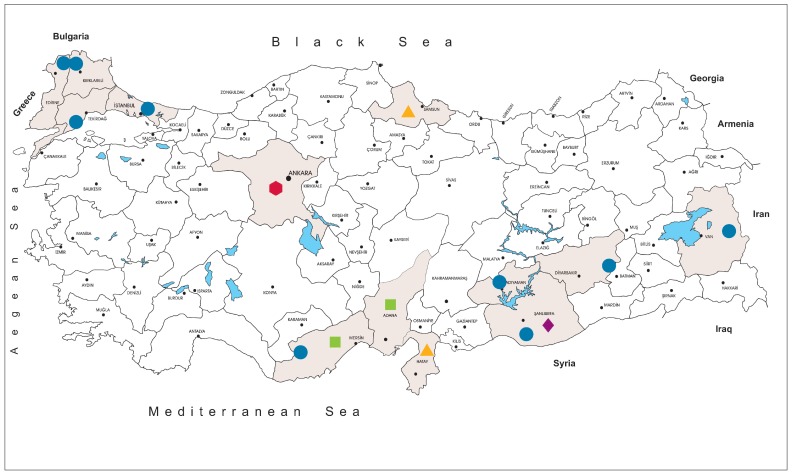
Illustrative map of the locations used for specimen collection in the study (circle: tick, triangle: avian, square: canine, diamond: bovine, hexagon: human).

The tick specimens were collected at 59 locations in Istanbul (northwest Anatolia, Thrace region), Edirne, Kırklareli and Tekirdağ (Thrace region), Mersin (southern Anatolia, Mediterranean region), Van (eastern Anatolia), Adıyaman, Diyarbakır and Şanlıurfa (southeastern Anatolia) provinces, between 2013–2018 (Figure 1). The specimens were removed from infested animals including cattle (*Bos taurus*), sheep (*Ovis aries*), goats (*Capra aegagrus hircus*) and dogs (*Canis familiaris*), or collected by flagging, using a 75 × 100 cm cloth over low and high vegetation. Upon collection, the ticks were stored in vials, transferred in dry ice, and identified morphologically to the species level using suitable taxonomic keys [22,23,24,25,26]. Identified ticks were pooled according to collection site, species and developmental stage, up to a maximum of 50 individuals per pool. The pools were disrupted by vortexing with 4.5 or 7.0 mm tungsten carbide beads (QIAgen, Hilden, Germany) in 500–700 μL of Eagle’s minimal essential medium (MEM), supplemented with 5% fetal bovine serum and 1% L-glutamine. The ground pools were centrifuged at 4000 rpm for 4 min and the supernatants were aliquoted and stored at −80 °C. High Pure Viral Nucleic Acid Kit (Roche Diagnostics, Mannheim, Germany) was used for nucleic acid purification, followed by complementary DNA synthesis, carried out using the High-Capacity cDNA Reverse Transcription Kit (Thermo Fisher Scientific, Hennigsdorf, Germany) with random hexamer priming, as described by the manufacturers.

### 2.3. Virus Screening

The detection of JMTV was performed by polymerase chain reaction (PCR). We used two primer sets, targeting the NS3-like protein gene, located on the genome segment 3 [1]. Having obtained the initial JMTV genome sequence, we updated the previously-described primers 317-5-126 and 317-3-383 as F: GTcACcGCcTCAGGcACaAA and R: 5′-tGAGGGcTGCAccTTcAGtA and employed both sets in individual reactions, with identical PCR conditions for screening [6,8]. Cell culture supernatants of the isolated JMTV strain (T36) were used as positive controls in the assays. Amplified products were visualized in a ChemiDoc XRS+ imaging system (Bio-Rad Laboratories, Munich, Germany), following electrophoresis in 1.5% agarose gels. The products were cleaned up using the PureLink PCR Purification Kit (Thermo Fisher Scientific, Hennigsdorf, Germany), and sequenced bi-directionally in an ABI PRISM 3500xL Dx Genetic Analyzer (Thermo Fisher Scientific, Hennigsdorf, Germany), using the BigDye Terminator v3.1 Cycle Sequencing Kit (Thermo Fisher Scientific, Hennigsdorf, Germany).

Specimens positive for JMTV nucleic acids were further screened for tick-borne phleboviruses, flaviviruses and nairoviruses using previously-described assays based on generic amplification. For phleboviruses, single-round amplification by the ppL1 and ppL2 primer sets, targeting the viral polymerase functional motifs located on the L-segment, were employed [27]. A nested PCR assay with degenerated primers targeting the NS5 conserved region was used for the flavivirus detection [28] Finally, screening for nairoviruses was carried out using a single-round PCR assay with primers amplifying the central motif A of the viral polymerase on the genomic L-segment [29]. CCHFV strain Ank-2 propagated in SW-13 cells, West Nile virus (WNV) NY99–4132 isolate propagated in Vero E6 cells and previously-identified phlebovirus-positive tick pools were used as controls. Detectable products from each protocol were cleaned up and sequenced as described above.

### 2.4. Animal and Human Specimens

Specimens from wild birds were collected at wildlife rehabilitation centers of the Ministry of forestry and water affairs in Samsun and Hatay provinces during necropsy. The centers are located in the convergence zone of the known bird migration routes from the Balkans and Asia to Africa over Anatolia. Canine sera were collected at local veterinary clinics and animal shelters during medical interventions by local veterinarians in Mersin and Adana provinces. Sera from cattle were collected from infested animals during tick removal in Şanlıurfa province. We further tested previously collected human specimens for JMTV infections. The specimens comprised sera from individuals with laboratory diagnosis of Crimean-Congo hemorrhagic fever, cerebrospinal fluid (CSF) and/or sera from individuals with febrile disease or meningoencephalitic symptoms of unknown etiology with/without tick bite history. The specimens were stored at −80 °C and available as aliquots of the original sample, purified RNA or cDNA, processed using a High Pure Viral Nucleic Acid Kit (Roche Diagnostics, Mannheim, Germany) and a RevertAid First Strand cDNA Synthesis Kit (Thermo Fisher Scientific). Nucleic acid purification and/or cDNA synthesis in unprocessed specimens were carried out as described for tick pools. All specimens were screened for JMTV using both sets of primers, as described above.

### 2.5. Virus Isolation

Processed tick homogenates were inoculated onto semi-confluent monolayers of African green monkey kidney (Vero E6, ATCC: CRL-1586) cells. The cells were obtained from the cell culture collection of the Department of Virology, Faculty of Veterinary Medicine, Ankara University. Briefly, the specimens (400–500 μL) were filtered through 0.22 μm sterile membrane filters (Merck Millipore, Darmstadt, Germany) and inoculated onto cells in T25 flasks (Nunc, Roskilde, Denmark), following dilution in equal volumes of Dulbecco’s modified Eagle’s medium (DMEM). Allowing cell adsorption for an hour, 5 mL of DMEM, supplemented with 5% fetal bovine serum, l-glutamine, 100 U/mL penicillin and 100 ug/mL streptomycin, was added. The cells were incubated in 5% CO_2_ atmosphere at 37 °C and monitored daily for cytopathic effects. Weekly blind passages were performed, and culture supernatants were tested for viral nucleic acids at each passage.

### 2.6. Next Generation Sequencing (NGS) and Data Analysis

A 100 μL aliquot of cell culture supernatants or tick pools was initially treated with Ambion DNase I and RNase Cocktail (Thermo Fisher Scientific, Hennigsdorf, Germany) and unencapsidated nucleic acids were removed using the Agencourt AMPure XP purification system (Beckman Coulter Biosciences, Krefeld, Germany). RNA was purified using the QIAmp Viral RNA Mini Kit (Qiagen, Hilden, Germany) and reverse transcribed into double-stranded cDNA using random hexamers SuperScript IV Reverse Transcriptase (Thermo Fisher Scientific, Hennigsdorf, Germany) and the NEBNext mRNA Second Strand Synthesis Module (New England Biolabs, Frankfurt am Main, Germany). The cDNA was cleaned up using the Agencourt AMPure XP reagent (Beckman Coulter Biosciences, Krefeld, Germany) and total yield and size distribution were optimized via the Agilent 2100 Bioanalyzer (Agilent Technologies, Waldbronn, Germany). Fragmentation, adaptor ligation and amplification were carried out using Nextera XT DNA Library Prep kit (Illumina, San Diego, CA, USA), using standard protocols provided by the manufacturer. The sequencing runs were performed in the paired-end mode, using the Illumina HiSeq 2000 (Illumina, San Diego, CA, USA).

The raw data were de-multiplexed and extracted in fastq format. Adaptor removal and trimming for quality and length (phred score of 33 and 30 base pairs (bp) minimum length) were carried out using Trimmomatic (version 0.35) [30]. Acquired reads were aligned to an in-house curated database, comprising Jingmen tick virus sequences deposited in the GenBank, using MALT (MEGAN alignment tool, version 0.3.8) and MEGAN (Metagenome Analyzer, version 6.12.3) [31,32]. Velvet (version 1.2.10) was employed to extract the aligned reads and contig assembly [33]. The contigs were mapped to the closely-related sequences and checked for heterogeneity by visual inspection using Geneious (version 11.1.5; Biomatters Ltd., Auckland, New Zealand). Gaps or ambiguities in the initial sequence were completed by PCR amplification using primers, designed on flanking regions, as necessary.

### 2.7. Phylogenetic and Structural Analyses

Obtained sequences were handled using Geneious (Biomatters Ltd., Auckland, New Zealand). BLASTn, BLASTn optimized for highly similar sequences (MEGABLAST) and BLASTp algorithms were used for nucleotide and deduced amino acid similarity searches in the public databases [34]. Nucleotide and putative amino acid alignments and pairwise sequence comparisons were generated via the CLUSTAL W [35]. Screening for recombination among JTV strains was carried out using algorithms implemented in the RDP4 software [36], in the default settings. SimPlot (version 3.5.1) was used for generating nucleotide similarity and bootscan plots [37]. Evolutionary history was inferred via the maximum-likelihood method based on the model estimated as the optimal substitution model individually for each alignment according to the Bayesian information criterion and conducted using MEGAX [38].

Protein domain and motif searches were performed using the NCBI conserved domain search tool (https://www.ncbi.nlm.nih.gov/Structure/cdd/wrpsb.cgi) and MOTIF Search (http://www.genome.jp/ tools/motif/) in the PFAM database [39,40]. Putative signal peptidase and protease cleavage sites were determined using SignalP (version 5.0; http://www.cbs.dtu.dk/services/SignalP-5.0/) and PROSPER (protease specificity prediction server; https://prosper.erc.monash.edu.au/home.html) [41,42]. Prediction of the membrane-spanning regions of the putative viral proteins were carried out by the TMpred program (https://embnet.vital-it.ch/software/TMPRED_form.html) [43]. NetNGlyc server (version 4.0; http://www.cbs.dtu.dk/services/NetOGlyc/) was used for identifying potential *N*-linked glycosylation sites [44].

## 3. Results

### 3.1. Isolation of the Prototype Jingmen Tick Virus (JMTV) Strain

The initial isolation of the JMTV strain T36 was carried out by the inoculation of a processed tick specimen onto Vero E6 cells. The specimen, a female *Rhipicephalus bursa* collected from sheep in Van province, was initially positive in generic nairovirus PCR during screening. Following cell culture inoculation, cytopathic effects, observed as cell elongation and vacuolization without prominent plaque formation, become visible, while no amplification could be attained in nairo-, flavi- or phlebo-virus assays. Finally, positive results and sequence confirmation were obtained using prototype JMTV primers, and cell culture supernatant of the second passage was used for genome sequencing.

### 3.2. JMTV Prevalence in Ticks

We collected and screened 7223 ticks, representing 5 genera and 15 species. The specimens originated from Kırklareli (*n* = 3443, 47.7%), Istanbul (*n* = 3044, 42.1%), Tekirdağ (*n* = 233, 3.2%), Mersin (*n* = 157, 2.2%), Edirne (*n* = 140, 1.9%), Diyarbakır (*n* = 78, 1.1%), Adıyaman (*n* = 68, 0.9%), Van (*n* = 36, 0.5%) and Şanlıurfa (*n* = 24, 0.3%) provinces in Turkey. Collections comprised adult ticks (female = 2541, 35.2%; male = 1639, 22.7%), larvae (*n* = 2094, 28.9%) and nymphs (*n* = 949, 13.1%) (Figure 1, Supplementary Table S1). The most frequently identified tick species was *Hyalomma marginatum* (*n* = 986, 13.7%), followed by *Hyalomma scupense* (*n* = 846, 11.7%), *Haemaphysalis parva* (*n* = 673, 9.3%) and others. The complete list of the tick cohort is provided in Supplementary Table S1.

The screening was performed in 630 tick pools and JMTV nucleic acids were detected in 25 (3.9%). Positive pools comprised *R. bursa* (7/25), *Rhipicephalus turanicus* (5/25), *H. marginatum* (4/25), *Hae. parva* (4/25), *Rhipicephalus sanguineus* sensu lato (3/25) and *Haemaphysalis inermis* (2/25) species, originating from Kırklareli (12/25), Mersin (5/25), Tekirdağ (4/25) and Van (4/25) provinces. Pools with detectable virus sequences comprised adult female and male ticks as well as nymphs (Table 1). We did not calculate species-based infection rates, as pool numbers for particular species were insufficient for a reliable prevalence assessment. No phlebo- or flavi-virus RNA could be detected in JMTV-positive pools. A single pool of R. bursa from Mersin province (T15) was positive in generic nairovirus PCR, further characterized as CCHFV by sequencing.

**Figure 2:**
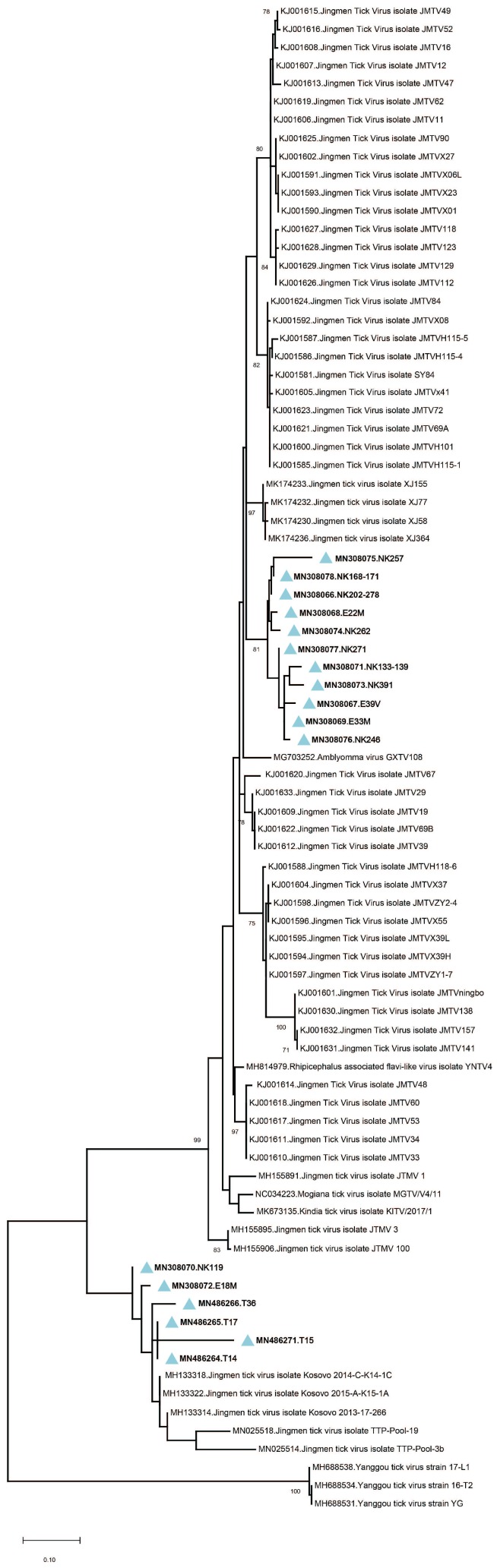
The maximum likelihood analysis of the JMTV partial segment 3 coding sequences (253 bp). The tree is constructed using the general time reversible (GTR) model, gamma distributed with invariant sites (G + I) for 500 replications. The sequences characterized in this study are given in bold and indicated with a blue triangle, GenBank accession number and specimen codes. Global virus strains are indicated by GenBank accession number, virus and strain/isolate name. Bootstrap values higher than 70 are provided.

Amplicon sequencing in tick pools with detectable JMTV provided 260-bp amplicons of the viral genome segment 3. The sequences showed up to 19.3% and 7% intramural diversity on nucleotide and putative amino acid levels, respectively. In the maximum likelihood analysis, they formed two well-demarcated clusters, with a shared common ancestor (Figure 2). The first cluster constituted a separate clade among JMTV strains of various origins, whereas the second remained distinct with strains detected in Kosovo and Trinidad and Tobago. No correlations of JMTV strains with either tick vector or geographical origin were observed in Anatolia.

### 3.3. JMTV in Human and Animal Specimens

We tested different patient cohorts for JMTV infections. One included 21 sera from individuals with febrile disease of unknown etiology (no tick bites) and 23 sera from individuals with a history of tick bites. Serum-CSF pairs from 16 patients with acute onset central nervous system infections without identifiable etiology were also evaluated. We further assessed sera from 27 CCHFV-infected individuals with viral loads of 9.8 × 10^3^ − 3.4 × 10^7^ genome copies. In total, 103 specimens from 87 individuals were screened, all with negative results for JMTV.

A number of tissues from avian species were screened for JMTV. The species included *Anas platyrhynchos* (mallard, *n* = 16), *Coturnix coturnix* (common quail, *n* = 9), *Perdix perdix* (gray partridge, *n* = 9), *Pernis apivorus* (European honey buzzard, *n* = 8), *Clanga pomarina* (lesser spotted eagle, *n* = 3), *Phoenicopterus roseus* (greater flamingo, *n* = 2), *Ardea herodias* (great blue heron, *n* = 1), *Ciconia ciconia* (white stork, *n* = 1), *Chroicocephalus ridibundus* (black-headed gull, *n* = 1), *Columba livia* (common pigeon, *n* = 1), *Corvus cornix* (hooded crow, *n* = 1) and *Pelecanus onocrotalus* (great white pelican, *n* = 1). The tissues comprised specimens from liver (*n* = 53), lung (*n* = 15), spleen (*n* = 7) and heart (*n* = 7). A total of 65 tissues from 53 birds were screened. Finally, canine sera from Mersin (*n* = 30) and Adana (*n* = 30) and bovine sera from Şanlıurfa province (*n* = 20) were assessed for JMTV. All non-tick specimens were negative.

### 3.4. Characterization of the JMTV Genomes

Direct NGS of the cell culture supernatant (T36) and PCR-positive tick pools (T14, T15, T17) provided total read numbers of 1.56–2.35 × 10^6^ with JMTV sequences comprising 1.3%–16.7% of the reads. The complete coding regions of the four genomic segments of the JMTV genome were obtained with incomplete non-coding ends, via NGS and Sanger sequencing (Table 2). Each genome segment comprised 2469–2940 nucleotides and alignment of the individual segments demonstrated maximum sequence diversity of 5.5% and 8% on nucleotide and amino acid levels, respectively (Supplementary Table S2). The sequences are stored in the GenBank with the accession numbers MN486256–MN486271.

**Figure 3:**
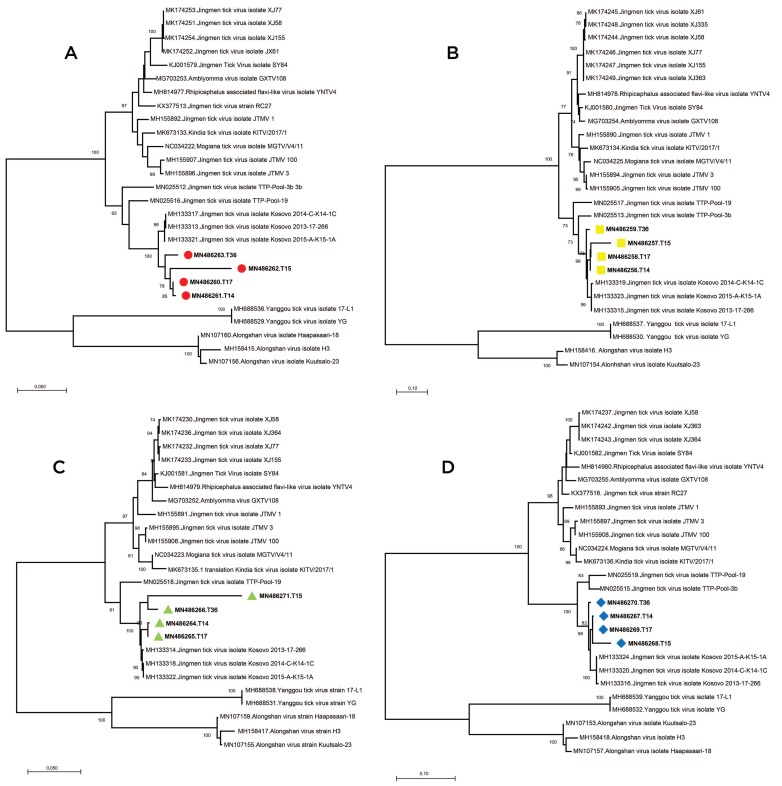
The maximum likelihood analysis of the JMTV complete coding sequences ((**A**): segment 1, (**B**): segment 2, (**C**): segment 3, (**D**): segment 4). The tree is constructed using the general time reversible (GTR) model, gamma distributed with invariant sites (G + I) for 500 replications. The sequences characterized in this study are given in bold and indicated with red circles (segment 1), yellow squares (segment 2), green triangles (segment 3) or blue diamonds (segments 4), GenBank accession number and specimen codes. Global virus strains are indicated by GenBank accession number, virus and strain/isolate name. Bootstrap values higher than 70 are provided.

We further analyzed the obtained sequences along with global JMTV genomes and closely related MGTV, ALSV, *Rhipicephalus*-associated flavi-like virus, *Amblyomma* virus, Kindia tick virus and Yanggou tick virus sequences (Supplementary Table S3). Alignment and maximum likelihood analysis of putative viral protein sequences encoded by individual genome segments revealed that viral genomes from Turkey are distinct from previously characterized viruses in China and Brazil (Figure 3). They formed a separate clade with strains detected in Kosovo, sharing a common ancestor with viruses identified in Trinidad and Tobaggo, similar to the phylogenetic relations observed by partial segment 3 analysis (Figure 2). A clustering pattern associated with geographical origin was noted for each JMTV genome segment, where multiple strains, particularly from China and Brazil, formed distinct clusters. Conversely, ALSV and Yanggou tick viruses, detected in particular tick species from China and Finland (17), remained distinct in each tree, practically serving as outliers (Figure 3).

**Figure 4:**
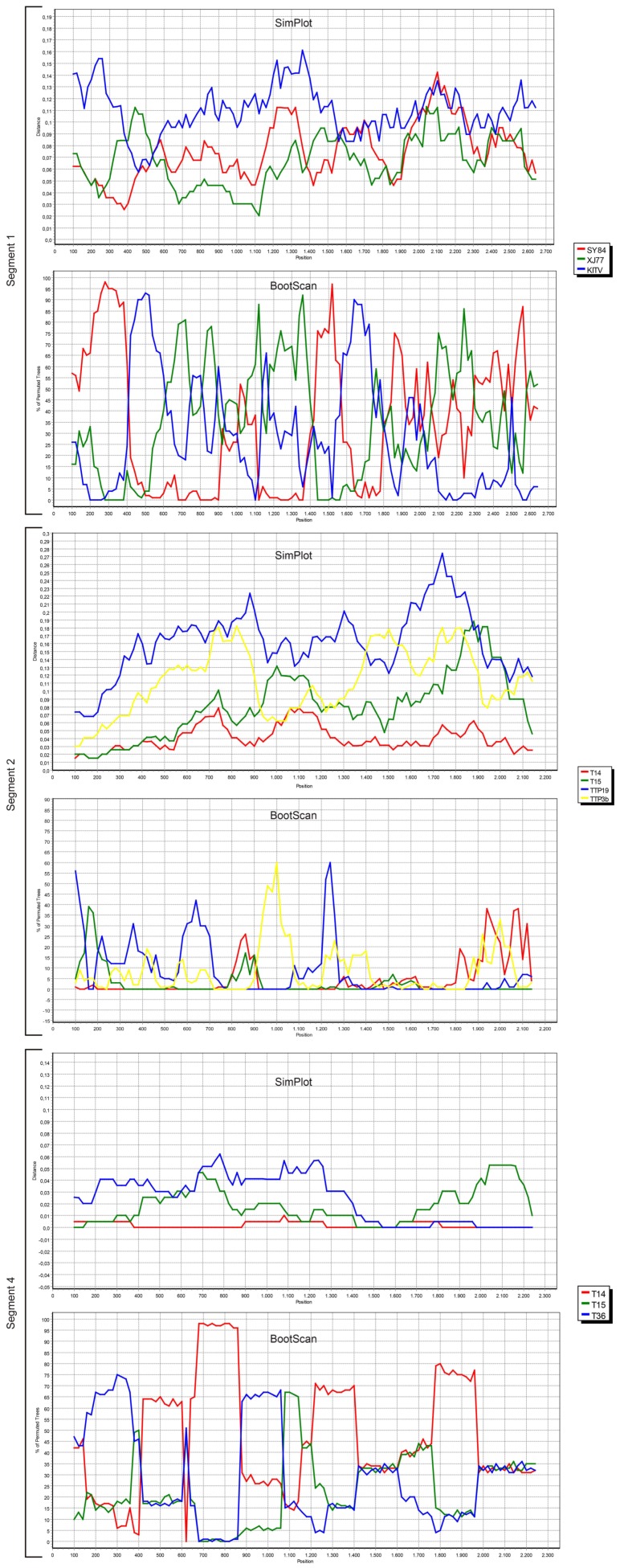
Similarity and bootscan plots of the alignments of the JMTV genome segments with evidence for recombination. The plots are prepared within a sliding window 200 base pairs (bp,) wide centered on the position plotted, with a 20 bp step size, for 1000 replications (GapStrip: On, Maximum Likelihood (Simplot), Neighbor-joining (Bootscan), T/t: 2.0). Virus strains used for analyses are indicated in the legends and in Supplementary File 3.

We also screened all publicly available JMTV coding regions for probable recombination events. No evidence of recombination was observed in segment 3, using RDP, Geneconv, Bootscan, MaxChi, Chimaera, SiScan, 3Seq tools in default settings. However, potential recombinations were identified in viral genome segments 1, 2 and 4 (Figure 4).

The most prominent event was documented in segment 4, where JMTV strains T14, T15 and T36 from Turkey were involved as parents or recombinants, identified by five of the tools used for screening. Potential breakpoints were located at positions 1707 and 2238 in the sequences, encoding for the putative viral membrane protein. Evidence for recombination was also observed in segment 2 among JMTV isolates T14 and T15 from Turkey, K14-1C from Kosovo, TTP-Pool-19 and TTP-Pool-3b from Trinidad and Tobaggo, *Rhipicephalus*-associated flavi-like virus, *Amblyomma* virus from China, via RDP, MaxChi, SiScan and 3Seq tools. Here, the recombination breakpoints were identified on positions 1572 and 1937 on the putative viral glycoprotein coding region. Potential recombinant and parent sequences were geographically segregated. Finally, segment 1 also seemed to have undergone recombinations, identified via MaxChi, Chimaera or SiScan tools, and involved JMTV strains SY84 and XJ77 as well as Amblyomma virus and Kindia tick virus, with breakpoint hotspots on positions 350 and 507.

### 3.5. Functional Analysis of the JMTV Genomes

**Figure 5:**
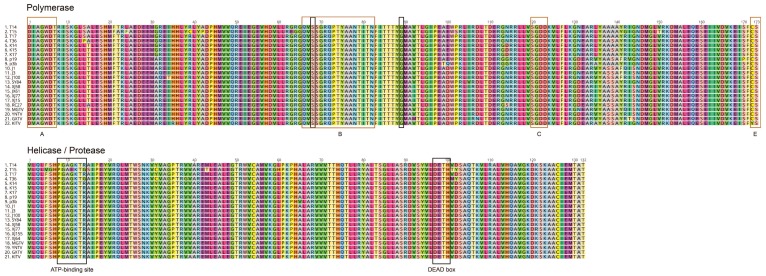
Alignment of the JMTV RNA-dependent, RNA polymerase and helicase/protease motifs, identified on viral genomic segments 1 and 3. The figures cover 576–748 and 341–472 residues of the mature nonstructural protein 1 (NSP1) and NSP2 proteins, respectively. Regions and residues corresponding to the conserved motifs of the flavivirus replicase (A, B, C, E) (brown) and with functional significance (black) are indicated. Information on virus strains used for comparison are provided in Supplementary Table S3.

The functional topology of genomic segments was retained in JMTVs and related viruses. Similar to the original isolates, genome segments 1, 2, and 3 are monocistronic and encode for single proteins, whereas segment 4 encodes for two overlapping polypeptides [5]. Segment 1 encodes for the nonstructural protein 1 (NSP1) in all strains, a 914-amino acid polypeptide with similarities to the flavivirus NS5 protein. The conserved motifs include a FtsJ-like methyltransferase domain (pfam01728), comprising 88 amino acids. This motif corresponds to the N-terminus of flaviviral NS5 protein and is involved in viral RNA capping [45]. It covers the positions 100–187 on the mature NSP1 and is conserved among JMTV strains with 89.7%–100% identities. The JMTV strains also comprise RNA-dependent RNA polymerase (RdRP) motif (pfam00972), forming a part of the flavivirus RNA-directed RNA polymerase (cd01699), encompassing 344–840 positions on the mature NSP1. A replicase active site as well as nucleic acid-nucleoside triphosphate binding sites, corresponding to conserved motifs of the flavivirus replicase, were identified (Figure 5). A membrane-spanning region, located on the 6th–25th residues on the NSP1 N-terminal, was also noted. The signal peptidase I recognition site was predicted to reside on 24th–25th residues (IEA-AS motif) in JMTV genomes from Turkey, Kosovo and Trinidad and Tobago.

The JMTV segment 2 encodes for the putative viral glycoprotein VP1. Interestingly, the size of the mature VP1 varies among JMTV and related strains. It comprises 744 amino acids in JMTV strains from Turkey, Kosovo, Trinidad and Tobago, 753 amino acids in viruses from Brazil and Guinea, and finally, 754 amino acids in other JMTVs. In viruses with 753–754 amino acid VP1, an identical nonapeptide motif (MTPLPQPST) is observed in the N-terminal. The mature VP1s contain 5–7 predicted transmembrane regions in various strains, with a potential signal peptidase cleavage site (GS(F)S-QE). The mature glycoprotein also carries two potential N-glycosylation sites (NTTF and NCTV) at varying positions (on residues 162 and 215 in JMTVs with 744 amino acid VP1).

Viral genome segment 3 encodes for the second putative nonstructural protein (NSP2), that comprise 808 amino acids, with sequence and functional similarities to the flavivirus NS2b–NS3 complex, with essential roles in viral polyprotein processing and genome replication [5]. NS3 is a member of the DEAD-like helicase superfamily (cl28899), a diverse family of proteins involved in ATP-dependent RNA or DNA unwinding. The DEXH-box helicase domain of NS3 protease-helicase comprises 132 amino acids in JMTV strains and resides on the 341–472. residues of the NSP2 (Figure 5). In this domain, the ATP-binding site is well-conserved among strains (PGAGKTR), except in T15 and T36, where it appears as PGAEKTG or PGAGKTG, respectively. The previously predicted protease cleavage site between functional NS2b-NS3 analogs is retained in all strains [5].

The fourth viral genome segment is distinct in the way that it is bicistronic and comprises two ORFs with overlapping reading frames (Table 2). The VP2 is the 254 amino acid putative capsid protein with a predicted signal peptidase cleavage site, located between residues 19–20, and a single N-glycosylation in all strains. The VP3 constitutes the putative viral membrane protein and comprises 505 amino acids in Kindia tick virus and JMTVs from Brazil and Trinidad and Tobago, while it comprises 538 amino acids in other JMTVs. Five or six potential N-glycosylation sites and 11–12 predicted transmembrane domains were recognized in VP3, according to the size of the mature protein. In MGTV and *Amblyomma* virus, as well as JMTV strains from Kosovo and Turkey, an adenylate kinase or related kinase motif (COG0563), involved in nucleotide transport and metabolism, was detected, encompassing residues 30–119 of the VP3. In addition, a predicted membrane protein motif (pfam09843, DUF2070) was detected in VP3 90–238th positions in MGTV, *Rhipicephalus*-associated flavi-like virus and JMTV strains from Kosovo and Turkey. These motifs are lacking in the remaining JMTV genomes.

## 4. Discussion

Following the isolation of the first local strain, we screened several cohorts for JMTV, aiming to explore virus activity in Anatolia and eastern Thrace. The tick specimens included questing or feeding specimens collected over five years from nine provinces from various regions, with diverse ecological niches (Figure 1). JMTV nucleic acids were detected in 3.9% of 609 pools (Table 1), collected from distinct regions in eastern Thrace, Mediterranean and eastern Anatolia. species form the genera *Rhipicephalus* and *Haemaphysalis* were infected, as well as *Hyalomma marginatum*, the main vector for CCHFV transmission in Turkey. Pairwise comparison of the partial segment 3 sequences showed significant diversity and the sequences grouped into two separate clades in phylogeny reconstruction, regardless of the host tick species or location (Figure 2). These data suggest a ubiquitous distribution of JMTV in ticks, with a propensity for widespread dispersion in Turkey. Similar infection of multiple tick species and considerable genetic variation in relatively confined areas were documented in China and Brazil [5,6,7,8,15].

JMTV was initially described in *Rhipicephalus microplus* ticks, and this species as well as *Rhipicephalus sanguineus* s.l. were shown to harbor virus strains in China, Brazil and Trinidad and Tobaggo [5,46]. Moreover, closely-related viruses MGTV and *Rhipicephalus*-associated flavi-like virus were detected in this species [6,7,8,9]. We detected JMTV RNA in all *Rhipicephalus* species in screened ticks in this study. Another related virus, Kindia tick virus, was identified in *Rhipicephalus geigyi* ticks in Guinea, Africa. These findings suggest that *Rhipicephalus sp.* ticks of the local fauna generally support the replication of JMTV-related viruses. JMTV has been detected in several additional tick species including *Amblyomma javanense*, *Dermacentor silvarum*, *Dermacentor nuttalli*, *Haemaphysalis campanulata, Haemaphysalis concinna*, *Haemaphysalis longicornis*, *Ixodes granulatus, Ixodes sinensis,* and *Ixodes persulcatus* ticks with varying prevalence rates, suggesting the lack of a species-specific restriction of viral replication [5,10,15]. We could not detect viral sequences in *Dermacentor or Ixodes* species, mainly comprising *Dermacentor marginatus* and *Ixodes ricinus* in our cohort. However, as our collection approach was mainly cross-sectional, the temporal aspects or the seasonality of the JMTV infection was not studied; therefore, infections in particular species might have escaped detection. Related strains ALSV and Yanggou tick virus were identified in *Ixodes persulcatus, Ixodes ricinus* and *D. nuttalli* ticks respectively [16,17], providing another example for particular tick species being infected with closely related Jingmenviruses. MGTV infection in various tick developmental stages has been documented [6], consistent with our detection of infected *H. marginatum* nymphs (Table 1). Overall, hard ticks from diverse genera could be infected with JMTV and related viruses and very likely to be vectors responsible for transmission to vertebrate hosts, with virus accumulation in tick salivary glands and midgut [6,15]. JMTV and ALSV RNA were also detected in *Aedes vexans*, *Anopheles yatsushiroensis*, *Armigeres* sp., *Culex pipiens* and *Cx. tritaeniorhynchus* mosquitoes, albeit with lower prevalence than in ticks and unknown implications for genetic exchange and transmission [5,16].

JMTV was initially isolated using invertebrate cell lines C6/36 and BME26 [5]. However, these cells were not proved to be consistently suitable for isolation or evaluation of plaque formation [6,7]. The embryo-derived tick cell line BME/CTVM23 was also successful in supporting JMTV replication, as well as experimental infections in *Amblyomma javanense* ticks [15]. We managed to isolate the prototype Anatolian strain using Vero E6 cells, deficient for type I interferon. Similarly, Vero cells were used for MGTV and ALSV isolations. The cell tropism of JMTV and related viruses as well as optimal cell line for virus propagation and subsequent phenotypic testing remains to be determined.

We sequenced and characterized four near-complete JMTV genomes, one from the original isolate and three from direct NGS on PCR positive tick pools. We further performed a comprehensive analysis of the coding regions of all available JMTV and related virus genomes. Genome topology and functional organization of the viruses from Anatolia were identical to the tick-associated Jingmen group viruses, with highest sequence identities to JMTV strains from Kosovo, in the Balkan peninsula. Phylogenetic reconstruction of individual genome segments further exhibited close relationships between the Kosova strains and the Anatolia strains (Figure 3). This is also evident in functional elements in the viral genome, as significant similarities in amino acid motifs were noted among these viruses. Comparison of the complete genomes also revealed a spatial segregation of viral subclades, suggesting local adaptive processes. This is further supported by the epidemiological data from infected ticks, where members of particular genera in the indigenous tick fauna were observed to harbor viruses in various regions, with considerable sequence diversity. These findings indicate that JMTVs are globally spread viruses, well-adapted to local tick populations.

A unique feature of the JMTV and related viruses is that they represent the first segmented, multicomponent (multipartite) viruses shown to produce animal infections [13]. Moreover, they form an evolutionary connection between segmented and unsegmented RNA virus genomes, with the prominent sequence homologies of two genome segments to flavivirus non-structural proteins [5,11]. The Jingmenvirus group include several diverse viruses detected in various insects and mosquitoes, as well as ticks [11,13]. In Guaico *Culex* virus, a mosquito-borne strain with five genome segments, presence of particular segment subsets seemed sufficient for transcription or replication, while complete genome was required for a full cycle of infection [13]. Similar mechanisms may be involved in the observed inconsistencies in cell culture susceptibility and host range of JMTVs. The genome of the JMTVs may also facilitate sequence exchange among strains, as multipartite viruses may undergo and display high frequencies of recombination and reassortment [47]. Despite indirect evidence of a probable reassortment event in Guaico *Culex* virus, these mechanisms have not been extensively studied or reported to contribute to the sequence diversity in tick-associated jingmen viruses [5,13]. Through analysis of all available sequences, we have identified probable recombination events in the JMTV genome. The potential recombinations mostly involved genetically related strains from Anatolia or China. The tick hosts provide a suitable background for genetic exchange among related viruses. Epidemiological data demonstrating distinct JMTVs in identical tick species suggest that co-infections might occur in nature. In our cohort, possible recombinants originated from identical tick genera. It is likely for these events to contribute to the evolution of genetically-related JMTV variants, such as *Rhipicephalus*-associated flavi-like virus or *Amblyomma* virus. Experimental infections in arthropod models will help to elucidate mechanisms and impact of genetic exchange in these viruses.

Among Jingmenviruses, JMTV and ALSV have been recently documented to cause human infections [15,16]. Initially, JMTV was detected in four human skin biopsy specimens from tick bite sites using NGS [15]. Subsequent screening revealed viremia in these individuals and additional patients were identified via serology. The clinical manifestations ranged from mild illness to severe disease requiring hospital admission. The disease presented with the non-specific symptoms of acute febrile disease and itchy or painful eschars at the site of tick bite, with or without lymphadenopathy. Similarly, ALSV was described in individuals with acute febrile disease comparable to the tick-borne encephalitis virus infections, without subsequent sequela [16]. The clinical presentation for infections by both viruses were non-specific and could not be readily distinguished from other tick-borne infections, such as anaplasmosis or rickettsiosis. In order to identify probable JMTV infections in Anatolia, we tested specimens from various patient cohorts, including acute febrile disease of unknown etiology with or without tick bites and those presenting with neurological symptoms. No JMTV amplification was attained in sera or CSF specimens from 60 individuals. Moreover, we screened 27 CCHFV patients with a wide range of viral loads, also without successful JMTV detection. In Kosovo, JMTV was observed to be present in three CCHFV-infected individuals, raising questions about the potential impact on CCHFV clinical presentation and progress [14]. This could not be replicated in our study, with a larger group available for testing. Lack of JMTV detection despite circulation in vectors in Anatolia may be due to several factors and result from sampling bias and variations in regional epidemiology. A similar observation was reported from an ALSV screening in Finland, where the virus was identified in *Ixodes ricinus* ticks but lacking in over 900 individuals with suspected tick-borne encephalitis, tested for viral nucleic acids as well as immunoglobulins [16]. Human JMTV infection is also not frequent in China, with a seroconversion rate of 1.6% (8 in 509) [15]. JMTV infections should be investigated prospectively in individuals with febrile diseases of unidentified etiology, especially in those with tick bites or risks for tick exposure, for reliable information on prevalence and epidemiology.

JMTV infections in vertebrates other than humans is likely, as supported by virus detection in primates, rodents and host-removed ticks [7,8,13,15]. Moreover, virus exposure in cattle has been documented, using in-house assays [5]. Currently, no evidence of symptomatic infections in livestock or companion animals is available. We could not detect viremic cattle or dogs, sampled in provinces with JMTV activity. However, we could not evaluate previous exposure by antibody testing, a limitation of the current study. Given the range of tick species that can be infected with JMTV and their feeding preferences, infections in a variety of vertebrates should be considered. However, a universal immunoassay with the ability to detect antibodies to circulating virus variants must be developed, for a reliable assessment of previous exposure in different vertebrate hosts.

Birds (class *Aves*) are involved in the transmission of several viruses, owing to the biodiversity, adaptive immune response enabling asymptomatic infections and opportunities for virus acquisition from various sources [48]. They may participate directly in the circulation of vector-borne viruses as amplification hosts, or act as dissemination vehicles for arthropod vectors [49]. Considered as one of the central elements in the ecological network of ticks, hosts and tick-borne pathogens, birds are involved in CCHFV and TBEV epidemiology, mobilizing infected *Ixodes*, *Hyalomma*, and *Haemaphysalis* ticks [49,50]. To investigate possible JMTV infections and their probable role in vector dispersion, we tested tissue specimens from 53 dead birds belonging to 12 species and could also not detect JMTV nucleic acids. JMTV viremia in avians, if occurring, might be short and without significant accumulation of virus particles in solid organs. Evaluation of exposure via antibody testing will provide an alternate approach to assess the involvement of avians in JMTV epidemiology.

In conclusion, we isolated for the first time, a JMTV strain in Anatolia, and carried out screening of ticks, canine, bovine and avian specimens, and people with clinical symptoms. Despite lack of detection in human and animal specimens, we identified JMTV in several tick species, including vectors of CCHFV. Moreover, we sequenced viruses originating from ticks and cell cultures. Structural and functional aspects of the genomes were similar to global JMTV isolates, with the highest identities to viruses from the Balkan Peninsula. We further provided evidence for genetic exchange among local JMTVs. JMTV must be considered in diagnosis of febrile diseases of unknown etiology.

## Figures and Tables

**Table 1:** Tick pools with detectable Jingmen tick virus (JMTV) sequences.

Location	Species	Composition	Host	Sequence Info
Mersin	*R. bursa*	2 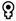 ,3 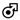	Goat	T14
Mersin	*R. bursa*	2 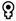 ,13 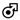	Goat	T15
Mersin	*R. sanguineus* s.l.	3 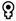 ,4 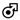	Dog	T17
Mersin	*R. sanguineus* s.l.	4 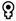	Dog	MN308068
Mersin	*R. sanguineus* s.l.	3 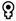 ,2 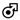	Dog	MN308072
Van	*R. bursa*	1 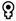	Sheep	T36
Van	*R. bursa*	1 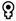	Goat	MN308069
Van	*R. bursa*	1 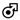	Goat	MN308066
Van	*R. bursa*	1 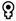	Sheep	MN308067
Kırklareli	*H. marginatum*	12 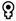	Cattle	MN308070
Kırklareli	*H. marginatum*	32 nymph	Cattle	MN308071
Kırklareli	*H. marginatum*	1 nymph	Cattle	MN308071
Kırklareli	*H. marginatum*	2 nymph	Cattle	MN308071
Kırklareli	*R. turanicus*	4 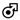	Cattle	MN308066
Kırklareli	*Hae. parva*	1 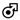	Cattle	MN308066
Kırklareli	*Hae. parva*	2 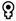	Cattle	MN308076
Kırklareli	*Hae. parva*	25 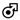	Cattle	MN308075
Kırklareli	*Hae. parva*	3 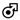	Cattle	MN308074
Kırklareli	*Hae. inermis*	5 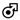	Cattle	MN308077
Kırklareli	*Hae. inermis*	1 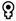	Cattle	MN308066
Kırklareli	*R. bursa*	7 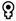	Cattle	MN308073
Tekirdağ	*R. turanicus*	25 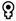	Cattle	MN308078
Tekirdağ	*R. turanicus*	20 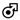	Cattle	MN308078
Tekirdağ	*R. turanicus*	27 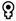	Cattle	MN308078
Tekirdağ	*R. turanicus*	13 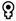	Cattle	MN308078

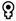
: Female, 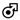: Male

**Table 2:** Organization of the JMTV genomes characterized in the study.

	Segment 1			Segment 2			Segment 3			Segment 4			
	5’	ORF	3’	5’	ORF	3’	5’	ORF	3’	5’	ORF1	ORF2	3’
T14	1–59	60–2804	2805–2940	1–146	147–2381	2382–2609	1–58	59–2485	2486–2650	1–121	122–886	856–2472	2473–2612
T15	1–54	55–2799	2800–2896	1–169	170–2404	2405–2469	1–89	90–2516	2517–2636	1–113	114–878	848–2464	2465–2574
T17	1–66	67–2811	2812–2894	1–173	174–2408	2409–2549	1–89	90–2516	2517–2643	1–105	106–870	840–2456	2457–2635
T36	1–86	87–2831	2832–2947	1–170	171–2405	2406–2556	1–97	98–2524	2525–2555	1–111	112–876	846–2462	2463–603

ORF: Open reading frame.

## Supplementary Materials

The following are available online at https://www.mdpi.com/1999-4915/11/11/1071/s1, Table S1: Distribution of tick species and pools according to the collection location, Table S2: Pairwise comparison of the nucleotide and amino acid sequences of the complete coding regions of the JMTV genome segments (segment 1), Table S3: Information on the global JMTV and related virus strains analyzed in the study.

## References

[1] Estrada-Pena A., de la Fuente J. (2014). The ecology of ticks and epidemiology of tick-borne viral diseases. Antivir. Res..

[2] Mansfield K.L., Jizhou L., Phipps L.P., Johnson N. (2017). Emerging tick-borne viruses in the twenty-first century. Front. Cell. Infect. Microbiol..

[3] Vandegrift K.J., Kapoor A. (2019). The ecology of new constituents of the tick virome and their relevance to public health. Viruses.

[4] Emanet N., Kar S., Dinçer E., Brinkmann A., Hacıoğlu S., Farzani T.A., Koçak Tufan Z., Polat P.F., Şahan A., Özkul A. (2019). Novel tick Phlebovirus genotypes lacking evidence for vertebrate infections in Anatolia and Thrace, Turkey. Viruses.

[5] Qin X.C., Shi M., Tian J.H., Lin X.D., Gao D.Y., He J.R., Wang J.B., Li C.X., Kang Y.J., Yu B. (2014). A tick-borne segmented RNA virus contains genome segments derived from unsegmented viral ancestors. Proc. Natl. Acad. Sci. USA.

[6] Maruyama S.R., Castro-Jorge L.A., Ribeiro J.M., Gardinassi L.G., Garcia G.R., Brandão L.G., Rodrigues A.R., Okada M.I., Abrão E.P., Ferreira B.R. (2014). Characterisation of divergent flavivirus NS3 and NS5 protein sequences detected in *Rhipicephalus microplus* ticks from Brazil. Mem. Inst. Oswaldo Cruz.

[7] Souza W.M., Fumagalli M.J., Torres Carrasco A.O., Romeiro M.F., Modha S., Seki M.C., Gheller J.M., Daffre S., Nunes M.R.T., Murcia P.R. (2018). Viral diversity of *Rhipicephalus microplus* parasitizing cattle in southern Brazil. Sci. Rep..

[8] Pascoal J.O., Siqueira S.M., Maia R.D.C., Juan Szabó M.P., Yokosawa J. (2019). Detection and molecular characterization of Mogiana tick virus (MGTV) in *Rhipicephalus microplus* collected from cattle in a savannah area, Uberlândia, Brazil. Ticks Tick Borne Dis..

[9] Villa E.C., Maruyama S.R., de Miranda-Santos I.K.F., Palacios G., Ladner J.T. (2017). Complete coding genome sequence for Mogiana tick virus, a Jingmenvirus isolated from ticks in Brazil. Genome Announc..

[10] Meng F., Ding M., Tan Z., Zhao Z., Xu L., Wu J., He B., Tu C. (2019). Virome analysis of tick-borne viruses in Heilongjiang Province, China. Ticks Tick Borne Dis..

[11] Shi M., Lin X.D., Vasilakis N., Tian J.H., Li C.X., Chen L.J., Eastwood G., Diao X.N., Chen M.H., Chen X. (2015). Divergent viruses discovered in arthropods and vertebrates revise the evolutionary history of the Flaviviridae and related viruses. J. Virol..

[12] Webster C.L., Waldron F.M., Robertson S., Crowson D., Ferrari G., Quintana J.F., Brouqui J.M., Bayne E.H., Longdon B., Buck A.H. (2015). The discovery, distribution, and evolution of viruses associated with *Drosophila melanogaster*. PLoS Biol..

[13] Ladner J.T., Wiley M.R., Beitzel B., Auguste A.J., Dupuis A.P., Lindquist M.E., Sibley S.D., Kota K.P., Fetterer D., Eastwood G. (2016). A multicomponent animal virus isolated from mosquitoes. Cell Host Microbe.

[14] Emmerich P., Jakupi X., von Possel R., Berisha L., Halili B., Günther S., Cadar D., Ahmeti S., Schmidt-Chanasit J. (2018). Viral metagenomics, genetic and evolutionary characteristics of Crimean-Congo hemorrhagic fever orthonairovirus in humans, Kosovo. Infect. Genet. Evol..

[15] Jia N., Liu H.B., Ni X.B., Bell-Sakyi L., Zheng Y.C., Song J.L., Li J., Jiang B.G., Wang Q., Sun Y. (2019). Emergence of human infection with Jingmen tick virus in China: A retrospective study. EBioMedicine.

[16] Wang Z.D., Wang B., Wei F., Han S.Z., Zhang L., Yang Z.T., Yan Y., Lv X.L., Li L., Wang S.C. (2019). A new segmented virus Associated with human febrile illness in China. N. Engl. J. Med..

[17] Kuivanen S., Levanov L., Kareinen L., Sironen T., Jääskeläinen A.J., Plyusnin I., Zakham F., Emmerich P., Schmidt-Chanasit J., Hepojoki J. (2019). Detection of novel tick-borne pathogen, Alongshan virus, in Ixodes ricinus ticks, south-eastern Finland, 2019. Euro Surveill..

[18] Bursali A., Keskin A., Tekin S. (2012). A review of the ticks (Acari: Ixodida) of Turkey: Species diversity, hosts and geographical distribution. Exp. Appl. Acarol..

[19] Inci A., Yildirim A., Duzlu O., Doganay M., Aksoy S. (2016). Tick-borne diseases in Turkey: A review based on one health perspective. PLoS Negl. Trop. Dis..

[20] Brinkmann A., Dinçer E., Polat C., Hekimoğlu O., Hacıoğlu S., Földes K., Özkul A., Öktem İ.M.A., Nitsche A., Ergünay K. (2018). A metagenomic survey identifies Tamdy orthonairovirus as well as divergent phlebo-, rhabdo-, chu- and flavi-like viruses in Anatolia, Turkey. Ticks Tick Borne Dis..

[21] Dinçer E., Brinkmann A., Hekimoğlu O., Hacıoğlu S., Földes K., Karapınar Z., Polat P.F., Oğuz B., Orunç Kılınç Ö., Hagedorn P. (2017). Generic amplification and next generation sequencing reveal Crimean-Congo hemorrhagic fever virus AP92-like strain and distinct tick phleboviruses in Anatolia, Turkey. Parasites Vectors.

[22] Filippova N.A. (1997). Fauna of Russia and neighbouring countries. Ixodid Ticks of Subfamily Amblyomminae.

[23] Walker J.B., Keirans J.E., Horak I.G. (2000). The Genus Rhipicephalus (Acari, Ixodidae): A Guide to the Brown Ticks of the World.

[24] Walker A.R., Bouattour A., Camicas J.L., Estrada-Pena A., Horak I.G., Latif A.A., Pegram R.G., Preston P.M. (2003). Ticks of Domestic Animals in Africa: A Guide to Identification of Species.

[25] Estrada-Peña A., Bouattour A., Camicas J.L., Walker A.R. (2004). Ticks of Domestic Animals in the Mediterranean Region.

[26] Apanaskevich D.A., Horak I.G. (2008). The genus *Hyalomma* Koch, 1844: V. Reevaluation of the taxonomic rank of taxa comprising the *H.* (*Euhyalomma*) *marginatum* Koch complex of species (Acari: Ixodidae) with redescription of all parasitic stages and notes on biology. Int. J. Acarol..

[27] Matsuno K., Weisend C., Kajihara M., Matysiak C., Williamson B.N., Simuunza M., Mweene A.S., Takada A., Tesh R.B., Ebihara H. (2015). Comprehensive molecular detection of tick-borne phleboviruses leads to the retrospective identification of taxonomically unassigned bunyaviruses and the discovery of a novel member of the genus Phlebovirus. J. Virol..

[28] Vázquez A., Sánchez-Seco M.P., Palacios G., Molero F., Reyes N., Ruiz S., Aranda C., Marqués E., Escosa R., Moreno J. (2012). Novel flaviviruses detected in different species of mosquitoes in Spain. Vector Borne Zoonotic Dis..

[29] Honig J.E., Osborne J.C., Nichol S.T. (2004). The high genetic variation of viruses of the genus Nairovirus reflects the diversity of their predominant tick hosts. Virology.

[30] Bolger A.M., Lohse M., Usadel B. (2014). Trimmomatic: A flexible trimmer for Illumina sequence data. Bioinformatics.

[31] Herbig A., Maixner F., Bos K.I., Zink A., Krause J., Huson D.H. (2016). MALT: Fast alignment and analysis of metagenomic DNA sequence data applied to the Tyrolean Iceman. bioRxiv.

[32] Huson D.H., Beier S., Flade I., Gorska A., El-Hadidi M., Mitra S., Ruscheweyh H.J., Tappu R. (2016). MEGAN community edition—İnteractive exploration and analysis of large-scale microbiome sequencing data. PLoS Comput. Biol..

[33] Zerbino D.R., Birney E. (2008). Velvet: Algorithms for de novo short read assembly using de Bruijn graphs. Genome Res..

[34] Altschul S.F., Gish W., Miller W., Myers E.W., Lipman D.J. (1990). Basic local alignment search tool. J. Mol. Biol..

[35] Thompson J.D., Higgins D.G., Gibson T.J. (1994). CLUSTAL W: Improving the sensitivity of progressive multiple sequence alignment through sequence weighting, position-specific gap penalties and weight matrix choice. Nucleic Acids Res..

[36] Martin D.P., Murrell B., Golden M., Khoosal A., Muhire B. (2015). RDP4: Detection and analysis of recombination patterns in virus genomes. Virus Evol..

[37] Lole K.S., Bollinger R.C., Paranjape R.S., Gadkari D., Kulkarni S.S., Novak N.G., Ingersoll R., Sheppard H.W., Ray S.C. (1999). Full-length human immunodeficiency virus type 1 genomes from subtype C-infected seroconverters in India, with evidence of intersubtype recombination. J. Virol..

[38] Kumar S., Stecher G., Li M., Knyaz C., Tamura K. (2018). MEGA X: Molecular evolutionary genetics analysis across computing platforms. Mol. Biol. Evol..

[39] Bateman A., Birney E., Cerruti L., Durbin R., Etwiller L., Eddy S.R., Griffiths-Jones S., Howe K.L., Marshall M., Sonnhammer E.L. (2002). The Pfam protein families database. Nucleic Acids Res..

[40] Marchler-Bauer A., Derbyshire M.K., Gonzales N.R., Lu S., Chitsaz F., Geer L.Y., Geer R.C., He J., Gwadz M., Hurwitz D.I. (2015). CDD: NCBI’s conserved domain database. Nucleic Acids Res..

[41] Petersen T.N., Brunak S., von Heijne G., Nielsen H. (2011). SignalP 4.0: Discriminating signal peptides from transmembrane regions. Nat. Methods.

[42] Song J., Tan H., Perry A.J., Akutsu T., Webb G.I., Whisstock J.C., Pike R.N. (2012). PROSPER: An integrated feature based tool for predicting protease substrate cleavage sites. PLoS ONE.

[43] Hofmann K., Stoffel W. (1993). TMbase—A database of membrane spanning proteins segments. Biol. Chem. Hoppe Seyler.

[44] Steentoft C., Vakhrushev S.Y., Joshi H.J., Kong Y., Vester-Christensen M.B., Schjoldager K.T., Lavrsen K., Dabelsteen S., Pedersen N.B., Marcos-Silva L. (2013). Precision mapping of the human O-GalNAc glycoproteome through SimpleCell technology. EMBO J..

[45] Koonin E.V. (1993). Computer-assisted identification of a putative methyltransferase domain in NS5 protein of flaviviruses and lambda 2 protein of reovirus. J. Gen. Virol..

[46] Sameroff S., Tokarz R., Charles R.A., Jain K., Oleynik A., Che X., Georges K., Carrington C.V., Lipkin W.I., Oura C. (2019). Viral Diversity of Tick Species Parasitizing Cattle and Dogs in Trinidad and Tobago. Sci. Rep..

[47] Varsani A., Lefeuvre P., Roumagnac P., Martin D. (2018). Notes on recombination and reassortment in multipartite/segmented viruses. Curr. Opin. Virol..

[48] Chan J.F., To K.K., Chen H., Yuen K.Y. (2015). Cross-species transmission and emergence of novel viruses from birds. Curr. Opin. Virol..

[49] de la Fuente J., Estrada-Pena A., Cabezas-Cruz A., Brey R. (2015). Flying ticks: Anciently evolved associations that constitute a risk of infectious disease spread. Parasites Vectors.

[50] Bente D.A., Forrester N.L., Watts D.M., McAuley A.J., Whitehouse C.A., Bray M. (2013). Crimean-Congo hemorrhagic fever: History, epidemiology, pathogenesis, clinical syndrome and genetic diversity. Antivir. Res..

